# Long-term nutrient addition increased CH_4_ emission from a bog through direct and indirect effects

**DOI:** 10.1038/s41598-018-22210-2

**Published:** 2018-03-01

**Authors:** Sari Juutinen, Tim R. Moore, Jill L. Bubier, Sini Arnkil, Elyn Humphreys, Brenden Marincak, Cameron Roy, Tuula Larmola

**Affiliations:** 1 Ecosystems and Environment Research Programme, Environmental Change Research Unit (ECRU), Faculty of Biological and Environmental Sciences, University of Helsinki, Viikinkaari 1, FI-00790 Helsinki, Finland; 2 Department of Geography, McGill University, 805 Sherbrooke St. W, Montreal, QC H3A 0B9 Canada; 3 Environmental Studies Department, Mount Holyoke College, 50 College Street, South Hadley, MA 01075 USA; 4 Department of Forest Sciences, University of Helsinki, Latokartanonkaari 7, 00790 Helsinki, Finland; 5 Department of Geography & Environmental Studies, Carleton University, 1125 Colonel By Dr, Ottawa, ON K1S 5B6 Canada; 6 Natural Resources Institute Finland, Latokartanonkaari 9, FI-00790 Helsinki, Finland

## Abstract

Peatlands are globally significant sources of atmospheric methane (CH_4_). While several studies have examined the effects of nutrient addition on CH_4_ dynamics, there are few long-term peatland fertilization experiments, which are needed to understand the aggregated effects of nutrient deposition on ecosystem functioning. We investigated responses of CH_4_ flux and production to long-term field treatments with three levels of N (1.6–6.4 g m^−2^ yr^−1^ as NH_4_NO_3_), potassium and phosphorus (PK, 5.0 g P and 6.3 g K m^−2^ yr^−1^ as KH_2_PO_4_), and NPK in a temperate bog. Methane fluxes were measured in the field from May to August in 2005 and 2015. In 2015 CH_4_ flux was higher in the NPK treatment with 16 years of 6.4 g N m^−2^ yr^−1^ than in the control (50.5 *vs*. 8.6 mg CH_4_ m^−2^ d^−1^). The increase in CH_4_ flux was associated with wetter conditions derived from peat subsidence. Incubation of peat samples, with and without short-term PK amendment, showed that potential CH_4_ production was enhanced in the PK treatments, both from field application and by amending the incubation. We suggest that changes in this bog ecosystem originate from long-term vegetation change, increased decomposition and direct nutrient effects on microbial dynamics.

## Introduction

**Figure 1:**
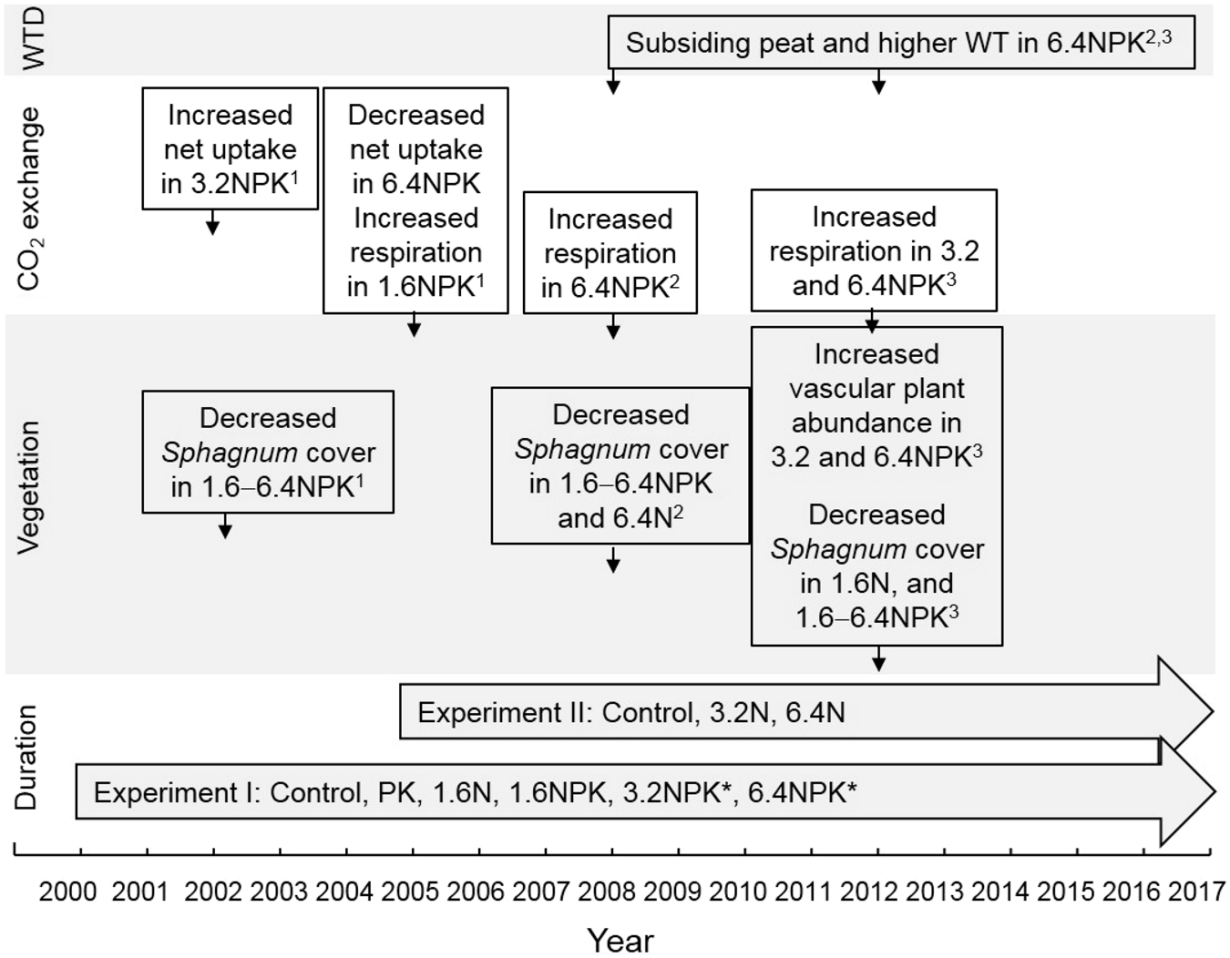
Time frame and major responses in vegetation, ecosystem CO_2_ exchange (net exchange, photosynthesis, and respiration), and water table relative to control in the fertilization experiments I and II at Mer Bleue bog. Vertical arrows indicate the years when a response was documented. *Treatments 3.2NPK and 6.4NPK were started in 2001, ^1^Bubier *et al*.^41^, ^2^Juutinen *et al*.^5^, ^3^Larmola *et al*.^7^.

Wetlands, including peatlands, are the largest single source of methane (CH_4_) to the atmosphere^1^. An increase in nutrient input, such as through atmospheric deposition, can alter the composition, biomass and productivity of vegetation and stimulate decomposition in peatlands^2–7^, potentially leading to changes in CH_4_ emission from peatlands to the atmosphere. Increased atmospheric nitrogen (N) deposition originates from gaseous N emissions from intensive farming, traffic, or industry^8^. Atmospheric deposition of phosphorus (P), the other limiting nutrient in peatlands, has increased as well, mainly originating from fertilized farmlands^9,10^. In addition, a change towards warmer and drier conditions can hasten mineralization rates leading to enhanced nutrient availability^11–14^. In this study, we tested the hypothesis that increased nutrient input elevates CH_4_ emission from nutrient-poor peatlands, arising from multiple changes in ecosystem functioning developed over the long-term (Fig. 1). These include changes in vegetation community, increased peat decomposition, peat subsidence and a higher water table (WT)^5,7^.

Peatlands vary in their nutrient status and hydrology. Bogs are the most nutrient-poor, because they depend on precipitation for water and nutrients due to their convex shape and surface above ground water inputs (ombrotrophy)^15,16^. Bogs, generally, have smaller CH_4_ fluxes to the atmosphere than more nutrient-rich peatlands, because of differences in vegetation, nutrients, and a lower WT^17,18^. A recent meta-analysis found that N enrichment, on average, doubled CH_4_ fluxes to the atmosphere across undrained wetlands, wet agricultural systems, and grasslands^19^. Responses of CH_4_ flux, however, to nutrient enrichments in peatlands have been mixed and often small^20–26^. Moreover, a seven year study found that responses of potential CH_4_ production and oxidation responded differently in the short and long-term^27^.

The emission of CH_4_ from peatlands is the balance between production of CH_4_ by methanogenic archaea which require anoxic conditions, and oxidation of CH_4_ by methanotrophic bacteria mainly in the oxic layer. When nutrient addition leads to increased CH_4_ efflux or production, this has often been attributed to the increased abundance and growth of vascular plants, specifically sedges (*Cyperaceae*), leading to more efficient gas transfer from the rooting zone to the atmosphere and an increased supply of fresh substrates to the methanogenic zone compared to moss-dominated vegetation^21–23^. Overall, better nutrient availability alleviates nutrient limitation of microbes and can increase both production and oxidation of CH_4_^20–33^. Methane efflux may also increase because of inhibition of CH_4_ consuming bacteria by NH_4_^+^ ^33^. On the other hand, NO_3_ can suppress CH_4_ production through competition for substrate by denitrification and it can be used as an oxidant increasing CH_4_ consumption, thus changing the emission rate^26^. Long-term field experiments may foster our understanding of aggregated effects of nutrient manipulation on ecosystem functioning. There are, however, few long-lasting fertilization experiments in peatlands. In one of these, an oligotrophic fen, increased potential CH_4_ production was attributed to vegetation change resulting in increased substrate supply and larger methanogenic population^21^.

We studied CH_4_ dynamics at long-term fertilization experiments at Mer Bleue bog, Ontario, Canada, where treatments with control and three levels of N as NH_4_NO_3_ with or without constant PK (KH_2_PO_4_) addition started in years 2000 (experiment I with N, PK, NPK) and 2005 (experiment II with N only) (Fig. 1). We measured CH_4_ fluxes between the ecosystem and the atmosphere weekly from May to August in 2005 (treatment years 1 and 6, for experiments I and II, respectively) and 2015 (treatment years 11 and 16). We incubated peat samples collected from the fertilization plots in 2016 to examine variations in the rates of potential CH_4_ production and consumption and supplemented this by examining the effect of laboratory PK addition on potential CH_4_ production. To quantify the peat subsidence visible through relative water table, we use spatially representative GPS data on peat surface elevation collected in years 2011 and 2013.

We hypothesized that 1) treatments with the highest long-term NPK loading would show the largest CH_4_ emission driven by a change from *Sphagnum* moss to dwarf shrub dominance associated with a faster litter decomposition rate and a rise in the WT; 2) potential CH_4_ production rates in peat samples from the field treatments would be higher in nutrient treatments with largest vegetation changes owing to reduced microbial nutrient limitation; and 3) potential CH_4_ production rates would increase with a laboratory PK amendment, with a larger response in the N-only field treatments than in NPK treatments, because of their nutrient limitation relative to the increased N supply.

## Study site and experimental set-up

The study was conducted in a long-term fertilization experiment at Mer Bleue bog, an ombrotrophic peatland complex covering 28 km^2^ in eastern Ontario, Canada (45.410017 N, 75.518348 W)^34^. The mean annual temperature (1971–2000) was 6.0 °C and the mean annual precipitation was 943 mm, of which 351 mm fell between May and August^35^. Estimated wet atmospheric deposition of N ranges from 0.5 to 0.8 g N m^−2^ yr^−1^ based on Canadian and US deposition observation networks (see also Turunen *et al*.^36^) and is small compared to the global N deposition gradient^8^.

The fertilization experiment is located in the northwestern part of the bog, where the surface has only a slight (20 to 30 cm) hummock and lawn topographic variation with hummocks comprising about 70% of the area^37^. The vascular vegetation is dominated by ericaceous shrubs *Chamaedaphne calyculata* (L.) Moench, *Rhododendron groenlandicum* (Oeder) Kron & Judd, *Kalmia angustifolia* L. and *Vaccinium myrtilloides* Michx. Graminoids and herbs are fewer in coverage, *Eriophorum vaginatum* L. and *Maianthemum trifolium* L. being the most common amongst those. The ground layer is dominated by mosses, mainly *Sphagnum capillifolium* (Ehrh.) Hedw., *Sphagnum magellanicum* Brid., and *Polytrichum strictum* Brid. Scattered trees, *Larix laricina* (Duroi) K. Koch., *Betula populifolia* Marshall and *Picea mariana* (Miller) BSP, grow in the area^38,39^. New species that have appeared in the long-term fertilized plots include the fern *Thelypteris palustris* (L.) A.Gray, and numerous moss species, for example *Pleurozium schreberi* (Brid.) Mitt. and *Aulacomnium palustre* (Hedw.) Schwägr^7^.

The fertilization experiment includes two sets of treatments^5,7,40–42^. The first experiment (I) was established in 2000-2001, which includes a control (C1), low level N-only, PK, and NPK additions at three N levels (1.6, 3.2 and 6.4 g N m^−2^ yr^−1^ as NH_4_NO_3_) (Fig. 1, Table 1). These levels range from about double the ambient N deposition at Mer Bleue to the high deposition rates occurring, for example, in Europe^8^. The second experiment (II) was started in 2005 to include a control (C2) and the 3.2 and 6.4 g N m^−2^ yr^−1^ levels without PK. PK treatment was applied at levels 0 and 5.0 g P and 6.3 g K m^−2^ yr^−1^ as KH_2_PO_4_ (Table 1). Each treatment has three plots, which are 3 m × 3 m and have at least 1 m buffer zone between them. The nutrients were added dissolved in distilled water corresponding to precipitation of 2 mm as seven applications every three weeks between May 1^st^ and August 31^st^. The controls received the same amount of distilled water.

**Table 1:** Treatments and addition of N, P, and K as NH_4_NO_3_ and KH_2_PO_4_.

Treatment	Start year	N (g m^−2^ yr^−1^)	P (g m^−2^ yr^−1^)	K (g m^−2^ yr^−1^)
Experiment I				
C1	2000	0	0	0
PK	2000	0	5	6.3
1.6 N	2000	1.6	0	0
1.6NPK	2000	1.6	5	6.3
3.2 NPK	2001	3.2	5	6.3
6.4 NPK	2001	6.4	5	6.3
Experiment II				
C2	2005	0	0	0
3.2N	2005	3.2	0	0
6.4N	2005	6.4	0	0

## Results

### Environmental variables, CH_4_ flux and surface elevation

**Figure 2:**
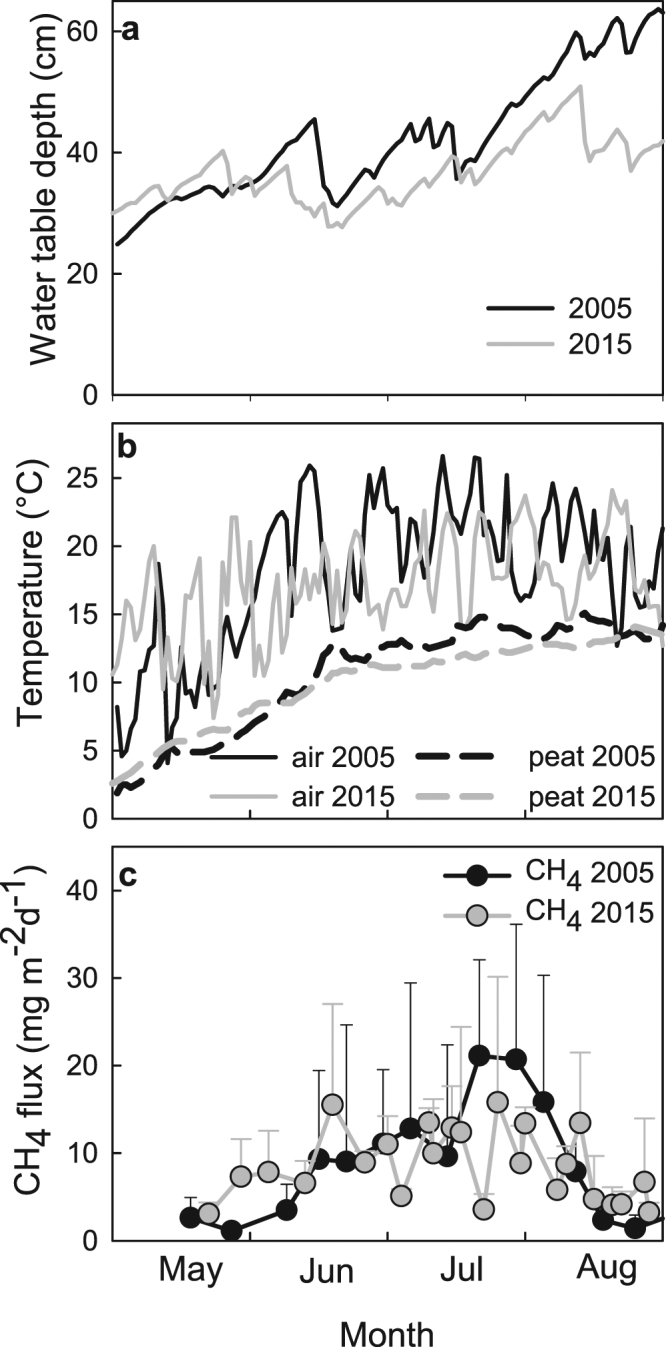
Patterns of WTD, temperature and CH_4_ flux, May to August 2005 and 2015. Daily averages of (**a**) WTD, (**b**) air and peat temperature at depth of 40 cm at the micrometeorological tower adjacent to the fertilization experiment, and (**c**) CH_4_ flux with standard error of mean in the control (C1) treatment.

We used a continuous series of water table depth (WTD) and air and peat temperatures measured at the micrometeorological tower site adjacent to the fertilization experiments to compare conditions from May 1 to August 31 in 2005 and 2015. The average WTD was slightly deeper in 2005 (43 cm) than in 2015 (37 cm) (Fig. 2a). The average air temperature was 18.0 °C in 2005 and 17.1 °C in 2015, and peat temperature at a depth of 40 cm was also slightly warmer in 2005 than in 2015 (10.7 vs. 10.1 °C, Fig. 2b). Following the typical seasonal pattern, the CH_4_ fluxes at the control plots (C1) rose from May onwards as the peat warmed and peaked in late July in both years, with a slightly larger seasonal peak in 2005 than in 2015 (Fig. 2c).

**Figure 3:**
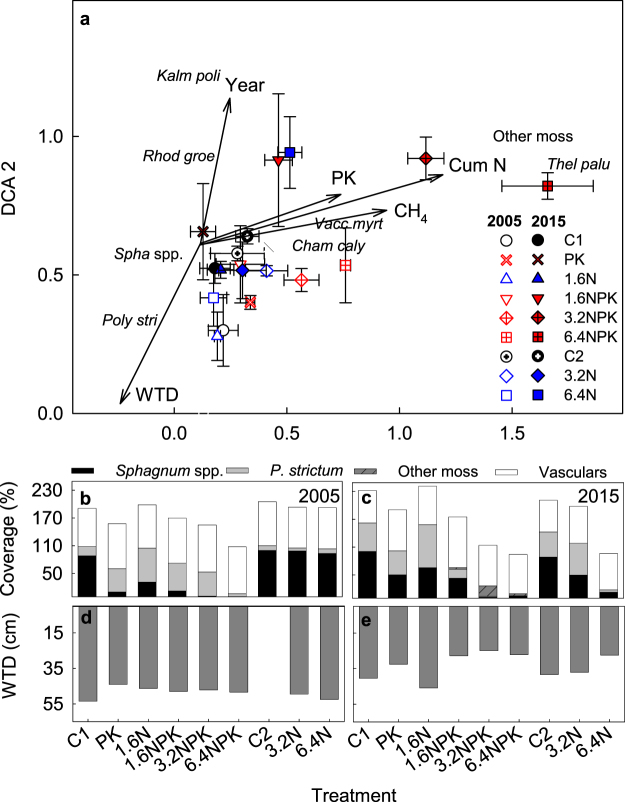
(**a**) DCA biplot of sample plots (treatment mean ± SE) with post-hoc fit of environmental variables. Treatments and years are distinguished with symbols and color. Axes 1 and 2 had eigenvalues of 0.27 and 0.15, and explained cumulatively 26.8% and 41.2% of the variation in the species data. Supplementary variables were cumulative nitrogen loading (Cum N), mean CH4 emission (CH_4_) PK fertlization, Water table depth (WTD) and year. (**b**,**c**) Mean cover of *Sphagnum*, *P*. *strictum*, other moss species, and vascular plants in 2005 and 2015 and (**d**,**e**) water table depth in the measurement points in 2005 and 2015.

Detrended correspondence analysis (DCA) revealed changes in plant community composition between treatments and between years 2005 and 2015 (Fig. 3). Plots in treatments 3.2NPK and 6.4 NPK diverged from the other plots in 2005 along the first axis, and this variation in plant community composition became larger by 2015. In addition, plots in treatments 1.6NPK and 6.4N moved along the first and second axes over the 10 year period. The first DCA axis represented gradients in cumulative N loading, PK addition and CH_4_ flux based on a *post-hoc* fit of environmental variables in species space. The second axis represented gradient in WTD and year. Methane flux correlated with cumulative N (*r* = 0.531), PK (*r* = 0.426) and WTD (*r* = −0.355), cumulative N loading correlated also with WTD (*r* = −0.557).

In 2005, the experiments had been exposed to treatments for 1 and 6 yr and *Sphagnum* moss cover decreased in all six year old nutrient addition treatments. In 2015, *Sphagnum* also decreased in the nutrient treatments started in 2005 (Fig. 3b,c), and abundances of both *Sphagnum* and *Polytrichum strictum* mosses correlated negatively with cumulative N load (Fig. 3b). An increase in vascular plants, mainly dwarf shrub abundance, was synchronous with a decrease in *Sphagnum* coverage, but with a smaller magnitude (Fig. 3b,c). Differences in WTD between the treatments were apparent only in 2015 when all NPK treatments (16 yr) and 6.4 N treatment (11 yr) had a WTD closer to the peat surface than the rest of the treatments (Fig. 3d,e).

The surface elevation showed effectively no change between 2011 and 2013 for the control plots where the average (±1 SD) difference was 1 ± 3 cm for C1 and 0 ± 2 cm for C2 (Supplementary information Table S1). The peat surface decreased, on average, by 5 ± 3 cm in the 6.4NPK plots and 6 ± 4 cm in the 6.4 N plots. In 2013, the average surface of the 6.4NPK plots was 13 cm lower than in C1 plots, and that of 6.4 N plots 5 cm below the surface of C2 plots.

### Treatment effects on CH_4_ flux

**Figure 4:**
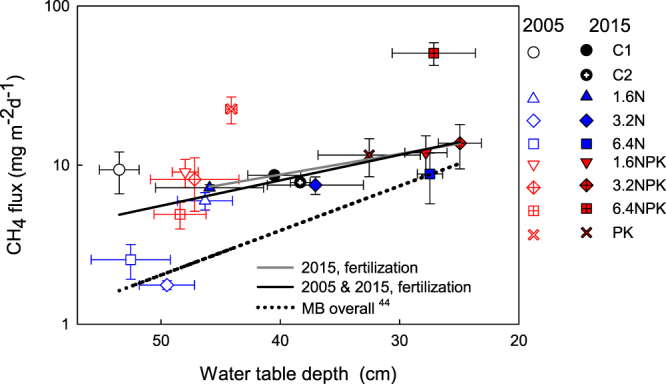
Treatment means of CH_4_ fluxes averaged over the May-August measurement period in 2005 and 2015 against treatment means of water table depth (±S.E., *n* = 3). Treatments and years are distinguished with symbols and color. Regression between CH_4_ flux and WTD was calculated using individual plots. Year 2015: log_10_CH_4_ = 1.461–0.013WTD, *R*^2^_*adj*_^.^ = 0.10, *p* = 0.061 and Year 2005 and 2015 combined: log_10_CH_4_ = 1.545–0.016WTD, *R*^2^_*adj*_ = 0.19, *p* < 0.001. Also shown is the regression for data outside the fertilization experiment at Mer Bleue bog: log_10_CH_4_ = 1.71–0.028WTD, *R*^2^_adj._ = 0.48, *p* < 0.05^54^.

Individual CH_4_ flux values were averaged for each plot over the study period (May-August) to compare treatments and control. In 2005, the mean CH_4_ flux was largest in the PK treatment (22.5 mg m^−2^ d^−1^), but with no significant treatment effects. In 2015, CH_4_ flux in the 6.4NPK treatment was large and differed significantly from the C1 (50.5 *vs*. 8.6 mg m^−2^ d^−1^, Tables 2 and 3, Fig. 4). In 2005, the relationship between CH_4_ flux and WTD was not significant, in part because of the small range in WTD: 22 cm among the 24 collars and 9 cm among the 8 treatments means (Fig. 4). In 2015, the relationship was significant with a wider range in WTD (42 cm among the 27 collars and 24 cm among the 9 treatments means) and the relationship was also significant when data from 2005 and 2015 were combined.

**Table 2:** Analysis of variance on the effects of year and treatment on the log_10_CH_4_ flux in experiments I and II.

Source	Experiment I			Experiment II		
	*df*	*F*	*p*	*df*	*F*	*p*
Corrected Model	12	3.839	0.003	5	4.896	0.019
Intercept	1	13.512	0.001	1	0.066	0.803
WTD*	1	0.482	0.494	1	0.751	0.409
Year	1	0.654	0.427	1	7.963	0.020
Treatment	5	1.625	0.193	2	0.178	0.840
Year × Treatment	5	4.706	0.004	1	0.004	0.953
Error	23			9		
Total	36			15		
Corrected Total	35			14		
R^2^_adj_	0.49			0.52		

*Covariate.

**Table 3:** Analysis of variance on the effects of treatments on log_10_CH_4_ flux in experiment I in years 2005 and 2015.

	*df*	2005		2015	
		*F*	*p*	*F*	*p*
Corrected Model	6	1.989	0.153	6.109	0.005
Intercept	1	0.362	0.560	29.165	<0.001
WTD*	1	0.282	0.606	2.167	0.169
Treatment	5	2.341	0.111	4.705	0.015
Error	11				
Total	18				
Corrected Total	17				
R^2^_adj_		0.26		0.64	

*Covariate.

### The effect of long-term field treatments and short-term PK amendment on CH_4_ production potential

**Figure 5:**
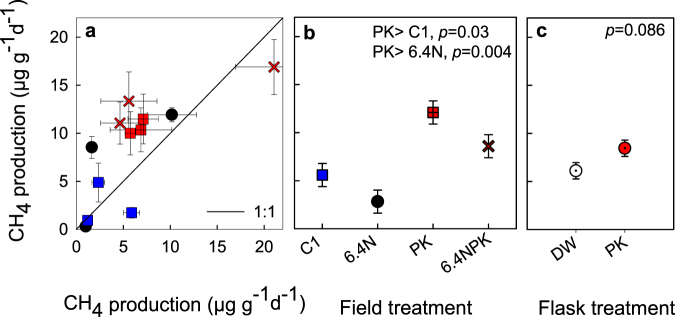
Anaerobic CH_4_ production potential. (**a**) Peat samples collected from the field-treatments incubated with distilled water (x-axis) plotted against samples incubated with a laboratory amendment of KH_2_PO_4_ (y-axis), symbol coloring as in Fig. 4. Data points represent the average of 4–6 analytical replicates per plot, with standard errors indicated. (**b**) Marginal means (±SE) showing the effects of field treatments and (**c**) flask amendment with PK on potential CH_4_ production (see Table 4), open symbol indicate treatment with distilled water (DW) and closed with PK solution (PK). Significant differences between treatments (Bonferroni multiple comparisons) are indicated in the b panel.

Sampling of peat relative to WTD in 2015 revealed that anaerobic CH_4_ production potentials in laboratory incubations were largest in samples closest to the WT (Supplementary Fig. 1). In 2016, peat samples collected from the C1, PK, 6.4 N and 6.4NPK field treatments at a depth of average WT position, had anaerobic CH_4_ production potentials ranging from 2 to 22 µg g^−1^ d^−1^ (Fig. 5a), with significantly larger rates in the long-term PK field treatments than in C1 and 6.4 N field treatments (Table 4, Fig. 5b). The laboratory PK amendment showed an increase in anaerobic CH_4_ production potentials across the field-treatments (*p* = 0.086, Table 4, Fig. 5c), with increases, relative to unamended samples, of 16%, 367%, 186% and 256% in the field treatments 6.4 N, C1, 6.4 NPK, and PK, respectively. The degree of decomposition of peat samples was higher by one unit in the Von Post scale in the peat cores of 3.2NPK, 6.4 NPK and 6.4N treatments relative to the controls.

**Table 4:** Three-way nested analysis of variance on the effects of treatment, plot nested within the treatment, PK amendment and the interaction treatment × PK amendment on potential CH_4_ production rates.

Source	df	F	p
Corrected Model	15	4.644	0.017
Intercept	1	147.01	<0.001
Plot (Treatment)	8	3.827	0.038
Treatment	3	11.001	0.003
PK amendment	1	3.824	0.086
Treatment × PK amendment	3	0.737	0.559
Error	8		
Total	24		
Corrected Total	23		
R^2^_adj_	0.7		

## Discussion

In agreement with our hypothesis, CH_4_ flux was nearly 5 times greater in the 6.4NPK treatment than in the control in the 16^th^ treatment year (2015), whereas differences were not significant in 2005. Conditions resulting in increased CH_4_ fluxes developed over the long-term and were detected only in the treatment with the longest and largest N loading with PK. The trends in vegetation, WTD, and peat subsidence in other NPK and 6.4N treatments, however, may indicate a similar response in the future. We interpret the response of CH_4_ flux to be the result of multiple environmental changes, initiated by a drastic decrease in *Sphagnum* moss abundance and concurrent increase in dwarf shrub biomass altering the quantity and quality of labile carbon input (Figs 1 and 3). These changes have led to increased ecosystem and heterotrophic respiration indicating stimulated decomposition^7,43^, and to peat subsidence and rise in the WT relative to the peat surface in the NPK plots (Fig. 4). The largest CH_4_ fluxes were associated with the highest WT positions, following the general pattern observed among and within peatlands^18^, but both the largest CH_4_ fluxes and highest WT position were associated with the largest additions of NPK.

Guo^44^ found a decrease in methanogen population size and no change in methanotroph populations in response to nutrient treatments at the depth of 30–40 cm in peat samples from the fertilization experiment collected in 2013. There were, however, broad changes in other microbial groups, for example a decrease in the fungi to bacteria ratio, and increased bacterial numbers and diversity in response to nutrient addition. The microbial results were similar to vegetation responses, in that NPK treatments induced stronger responses than N alone due to NP co-limitation at the site^45^. Vegetation change itself can alter microbial community composition and CH_4_ production potential as shown by Robroek *et al*.^46^. The results of their vegetation manipulation study, contrary to ours, showed that an intact *Sphagnum* layer with graminoid and ericoid removal resulted in a decrease in CH_4_ production potential by reducing root derived substrates.

In our study, both the long-term field and short-term lab addition of PK were associated with larger CH_4_ production potential than control and N-only treatments (Table 4, Fig. 5). This may be due to better availability of suitable substrates driven by increased plant productivity, changes in litter quality, and stimulation of decomposition^7,43,46^. The immediate increase of CH_4_ production potential in response to PK amendments suggests a direct positive nutrient impact on the microbial community, relieving methanogenic and syntrophic microbes from nutrient deficiency. This result agrees with Pinsonneault *et al*.^43^ who found increased activity of β-D-glucosidase enzyme with NPK fertilization at Mer Bleue bog. We cannot distinguish whether methanotrophs and CH_4_ consumption were inhibited by higher concentrations of NH_4_ owing to its similarity with CH_4_ and inhibition of the enzyme responsible for CH_4_ oxidation^20,27,31^.

Previous data on CH_4_ production from fertilization experiments varying in site characteristics and experimental length show mixed results, illustrating that methanogenic responses to nutrients are complex. Keller *et al*.^27^ found in short-term and 6 yr-long treatments that P and NP (NH_4_, up to 10 g N m^−2^ yr^−1^ and 2 g P m^−2^ yr^−1^) stimulated CH_4_ production in a rich fen, but inhibited it in a nutrient poor bog, suggesting that initial conditions, including nutrient availability, may determine the response. Eriksson *et al*.^21^ inferred from long-term data that the CH_4_ responses to nutrients are dependent on the WTD and vegetation, and are related to the amount, development and nature of microbial populations. Site properties, thus, may determine whether nitrate and other N oxides decrease CH_4_ production in N-only treatments if methanogenesis becomes outcompeted by energetically more efficient terminal electron acceptors and competition for substrate by denitrification^26,47,48^.

Mer Bleue, a bog dominated by dwarf shrubs and mosses with a deep WT, has not exhibited a shift to graminoid vegetation associated with an increase in CH_4_ fluxes as in some other N addition experiments. In a boreal ombrotrophic bog, 6 years of NH_4_NO_3_ addition (10 g N m^−2^ yr^−1^) resulted in a doubling of CH_4_ emission, attributed to the increase in sedge *Eriophorum vaginatum* abundance^23^. In Degerö Stormyr, an oligotrophic fen in Sweden, N addition decreased CH_4_ fluxes in plots with a high sedge cover, but increased fluxes slightly in plots with a small sedge cover; this was interpreted as being caused by changes in the distribution of roots and consequent effects on substrate supply^49^. Later in the same experiment, 12 years of fertilization with 3 g N m^−2^ yr^−1^ increased potential CH_4_ production significantly in the layer of maximal CH_4_ production near the water table. This was explained by a marked increase in the abundance of sedge vegetation and increases in the amount of labile substrate from root exudation^21^. Similar to our treatments with N-only, no or very small effects have been found in short- to mid-term NH_4_ or NH_4_NO_3_ addition experiments in oligotrophic fens^22,24,50^.

In conclusion, elevated CH_4_ fluxes at Mer Bleue are the result of several ecosystem processes altered by 16 yr of fertilization at a rate of 6.4 g N m^−2^ yr^−1^, plus P and K. The long-term addition of NPK has affected microbial community structure^44^ and enzyme activities^43^ indirectly through changes in vegetation and peat properties. Wetter conditions resulting from loss of moss, increased decomposition and peat subsidence in the highest NPK treatment have moved the zone of CH_4_ production closer to the peat surface, thus increasing the availability of added nutrients and substrates to methanogens, and likely reducing the proportion of CH_4_ consumption. In addition, PK addition seems to stimulate CH_4_ production directly, while the addition of NH_4_NO_3_ alone has had little direct effect on either CH_4_ production or consumption. However, comparison of this study with the literature shows that responses of CH_4_ dynamics are not universal across peatland ecosystems and thus merits further long-term investigation of multiple processes in the ecosystem.

## Methods

### Methane flux measurements

Methane fluxes were measured approximately weekly from the end of May to the end of August in 2005 and 2015 using a non-steady state, static, closed chamber method employing permanent collars with an area of 0.045 m^2^ inserted into the peat to a depth of 15–20 cm in each of the treatment plots. We used opaque chambers made of polycarbonate bottles (volume 18 L), which were covered with aluminum foil to reduce heating. We randomized the order in which the plots were measured between 8 am and 4 pm. In total 15 and 24 measurements per collar were made in 2005 and 2015, respectively; regardless of these differences, they were equally distributed over the May through August measurement period and the means should not be biased.

The top of the collars were grooved and were carefully cleaned of litter and filled with water to ensure airtight conditions before placing the chamber. Chamber headspace air was sampled 6 times at 0, 5, 10, 15, 20, and 30 min. Air samples (30 mL) were withdrawn through tubing and a three-way stopcock after mixing the air in the chamber and tubes by pumping with a 60 mL plastic syringe. The CH_4_ concentration was determined within a day after collection in 2005. In 2015, the air sample was injected into a 12 mL pre-evacuated glass vial and analyzed within 1–2 weeks. Methane concentrations were analyzed using a Shimadzu Mini II gas chromatograph (Shimadzu, Kyoto, Japan) and Varian CP-3800 gas chromatograph (Varian, Inc., Walnut Creek, CA, USA) in 2005 and 2015, respectively, both equipped with a packed column (Poropak Q column with 80/100 mesh, Alltech, 8 Deerfield IL, USA), flame ionization detector (FID) SRI-310C, with column and detector temperature of 50 °C and 110 °C, respectively. The gas chromatographs were calibrated for CH_4_ with multiple standards (0–22 ppm).

Fluxes of CH_4_ were calculated from the slope of the linear regression of gas concentration in the chamber headspace against time, chamber volume corrected for collar volume, surface area, and chamber temperature and ambient pressure. Each measurement series was checked visually by plotting CH_4_ concentration against time to check for leakage, ebullition or saturating CH_4_ concentration and unrealistically large initial CH_4_ concentration. Less than 5% of measurements were discarded and the measurements retained for analysis were associated with time series regression coefficients of determination of 0.90 or greater if there was an increase or decrease in the concentration. This rule was not followed in the case of near zero fluxes, *i*.*e*. when there was no change in the concentration over the time.

### Characterization of surface elevation, water table position, temperature and vegetation

We had observed the 6.4 NPK plots to be wetter than surrounding plots and to have subsiding peat surfaces^5,7^. To quantify these changes, a systematic survey of surface elevation was conducted in a subset of treatments (C1, C2, 6.4NPK and 6.4N) in 2011 and 2013. Calibration points as well as precise location points and their respective elevation above sea level were measured in treatment plots using a differential global positioning systems (DGPS) GRX-1 (Sokkia Corp, Mississauga, Ontario) and Trimble 5800 (Trimble Navigation Ltd, Dayton, Ohio). The plots were surveyed from a movable ladder set between the boardwalk and a fence with no disturbance to the peat and vegetation. The DGPS was attached to a levelled rod with a piece of plywood to gently keep it at the moss surface for measuring the elevation of the peat or *Sphagnum* moss surface. The systems were referenced to a base station that was positioned within 500 m from the surveying area on stable ground or over a Natural Resources Canada monument and permanent benchmark points near the plots were used to calibrate between the two years. Location and elevation above the sea level were recorded on approximately 220 grid points per plot in 2011. In 2013, approximately 104 grid points were measured in C1 and 6.4 NPK plots and 33 grid points were measured in the less spatially variable C2 and 6.4N plots. The horizontal and vertical instrumental precision were ±0.01 m and ±0.015 m (GRX-1, Sokkia) and ±0.01 m and ±0.02 m (Trimble 5800), respectively. ArcMap v10.1 (ESRI, 2013) was used to create surface rasters for the 2011 and 2013 years. The difference between raster surfaces for these two years was used to identify the change in elevation within the plots.

Water table depth (WTD) and chamber temperature were measured concurrent with CH_4_ flux measurements. Water table position was measured by determining its distance from the peat surface in perforated 2 cm diameter tubes that were inserted next to each collar. To characterize the year-to-year differences in conditions affecting CH_4_ fluxes, we also used continuous air and peat temperature and WTD measurements, averaged to daily values, made at the eddy covariance flux tower 100 m south of the fertilization plots. The abundance of vegetation and species composition was characterized in 60 × 60 cm quadrats inside the treatment plots by visually estimating the % cover of species (2005) and using the point intercept method (2015)^51^. The point intercept values were scaled to % values. We present here data measured in July, representing approximately peak above-ground biomass.

### Potential CH_4_ production rates

We conducted two laboratory incubations of peat collected from the fertilization plots to identify 1) the effect of sample location relative to WT on potential CH_4_ anaerobic production and aerobic consumption rates across all field treatments, and 2) the effect of selected field treatments (C1, C2, PK, 6.4 N, and 6.4NPK) and laboratory amendment with PK on potential anaerobic CH_4_ production. The first incubation experiment was a pre-study to help focus sampling for the second incubation experiment and, as it showed a potential PK effect, we conceived the second incubation experiment. For the first incubations one 40 cm long 10 cm × 10 cm square peat core was collected from each plot in May 2015 and split into 10 cm sections. The depth from the surface and the distance of each section to the WT at the time of sampling was recorded (at the main flux tower weather station, water table was about 33 cm below the hummock surface). The samples were stored at 5 °C prior to incubation that took place in June 2015.

Approximately 4 to 5 samples per treatment, totaling 38 and 48 samples, were selected for anaerobic and aerobic incubations, respectively. Three subsamples of ~12.5 g of wet peat from the each 10 cm sections were placed in 125 mL Erlenmeyer flasks for both the anaerobic and aerobic incubations and stored in the dark at 20 °C^52^. The anaerobic set was purged with Ultra High Purity nitrogen (N_2_) for 1 h to ensure anoxic conditions, capped with a rubber SubaSeal stopper and incubated for 21 d. Flask headspace was sampled (3 mL) immediately after purging and every third day, the removed gas being replaced with N_2_. For aerobic CH_4_ consumption potential, 125 μL of pure CH_4_ was added to ambient air in the flask. Flask headspace was sampled with a 3 mL syringe initially and after 1, 3, and 5 d, being replaced by ambient air. Methane concentrations were determined within a few hours of sampling on a Shimadzu Mini II gas chromatograph as described above. Rates of potential CH_4_ production and consumption were calculated from changes in headspace CH_4_ concentration, accounting for changes associated with dilution over time, and expressed per mass of dry peat, determined by oven drying peat samples at 50 °C at the end of the incubation. The first incubation study showed that greatest CH_4_ production and consumption potential rates were from peat sections just below and at the WT, respectively (Supplementary information Figure S1).

For the second incubation study we sampled the C1, PK, 6.4 N, and 6.4NPK treatments in July 2016 when the WT was 20–60 cm below the surface of the fertilization plots. One sample (10 × 10 × 10 cm) from each treatment plot was collected at the WT, the zone of maximum CH_4_ production according to pre-study, and divided into 10 subsamples of 12.5 g for anaerobic incubation (prepared as described above). One subsample from each plot was oven-dried to estimate the moisture content. Five subsamples were amended with PK, by adding 100 μg of P per gram of dry soil, dissolved in water as 0.439 g L^−1^ KH_2_PO_4_ and another five subsamples were treated with deionized water as a control. The flasks were sampled for headspace CH_4_ concentration over a 12 d incubation (as the results from the pre-study indicated no change in trend for CH_4_ production after 10–12 d), oven dried afterwards and production rate calculated as described above.

### Data analyses

To characterize the relationships between mean CH_4_ flux, plant community composition, cumulative N loading and WTD among the fertilization plots, we performed detrended correspondence analysis (DCA) with *post-hoc* fit of year and environmental variables as supplementary variables. A common analysis for both years was performed by transforming the 2015 vegetation data into percentage values. The DCA was performed on log_10_-transformed centered plant species data using Canoco 5^53^.

Treatment effects on surface topography were assessed by comparing the surface rasters of elevation between years 2011 and 2013 in treatments 6.4 NPK, 6.4 N, C1 and C2 (*n* = 3 per treatment). Seasonal means of daily CH_4_ fluxes and WT were calculated for each plot (*n* = 3 per treatment, two collars in one of the 6.4NPK plots were first averaged for each day), and used in regression analysis to examine the relationship between WT and CH_4_ flux. Log_10_-transformed data were used to fit linear regression allowing a comparison with that for overall Mer Bleue data from Moore *et al*.^54^. Treatment effects on CH_4_ fluxes were analyzed using analysis of variance, handling the two experiments separately. First, treatment and year were used as fixed factors and WT as covariate, and second, when there was significant year × treatment interaction the data were analyzed by years. Differences between the control and treatments were assessed by Dunnett’s t *post hoc* test. Data were log_10_ transformed to meet the requirement of equal variances and normal distribution.

Effects of long-term fertilization treatments (control, 6.4N, PK, and 6.4NPK) and laboratory amendment of PK on potential production of CH_4_ were analyzed using three-way nested ANOVA with Treatment, Plot nested within the Treatment, and PK amendment as the three factors and the Treatment × PK amendment interaction was also included. Treatment differences were assessed using pairwise Bonferroni comparisons. Five laboratory replicates for each plot and treatment were averaged prior to the analyses (treatment *n* = 3).

### Data Availability

The data set generated during the current study will be available through Mount Holyoke College Institutional Archive.

## Electronic supplementary material

Supplementary Information
