# Supplementary material for: Long-term nutrient addition increased CH4 emission from a bog through direct and indirect effects
Source: Sci Rep. 2018 Mar 1;8:3838. doi: 10.1038/s41598-018-22210-2 (PMC5832806; doi:10.1038/s41598-018-22210-2)
Supplement: Supplementary file 1 — Supplementary Information [file 41598_2018_22210_MOESM1_ESM.pdf]

Long-term nutrient addition increased CH_4 emission from a bog through direct and indirect effects

Sari Juutinen, Tim R. Moore, Jill L. Bubier, Sini Arnkil, Elyn Humphreys, Brenden Marincak, Cameron Roy, and Tuula Larmola

Supplementary information

Supplementary information Table S1

Mean surface elevation in the treatment plots in 2013, with standard deviation (SD) and treatment mean of elevation difference between control and treatment and treatment means of the change in elevation between 2011 and 2013.

Treatment, plot	Treatment year (2013)	Plot mean, elevation in 2013 (m a.s.l.)	SD (cm)	Treatment mean, elevation (m a.s.l.)	Difference, control-treatment (cm)	Mean change 2011-2013 (cm)
C1 a	14th	70.7503	4.6			
C1 b	14th	70.7338	7.2	70.7324		+1.4
C1 c	14th	70.7130	5.6		13.1	
6.4NPK a	13th	70.6046	7.7			
6.4NPK b	13th	70.5631	9.4	70.6014		-5.1
6.4NPK c	13th	70.6365	7.9			
C2 a	9th	70.7423	5.2			
C2 b	9th	70.7312	3.2	70.7476		0
C2 c	9th	70.7694	4.9		5.1	
6.4N a	9th	70.7179	8.3			
6.4N b	9th	70.7248	7.7	70.6962		-5.7
6.4N c	9th	70.6460	5.8			

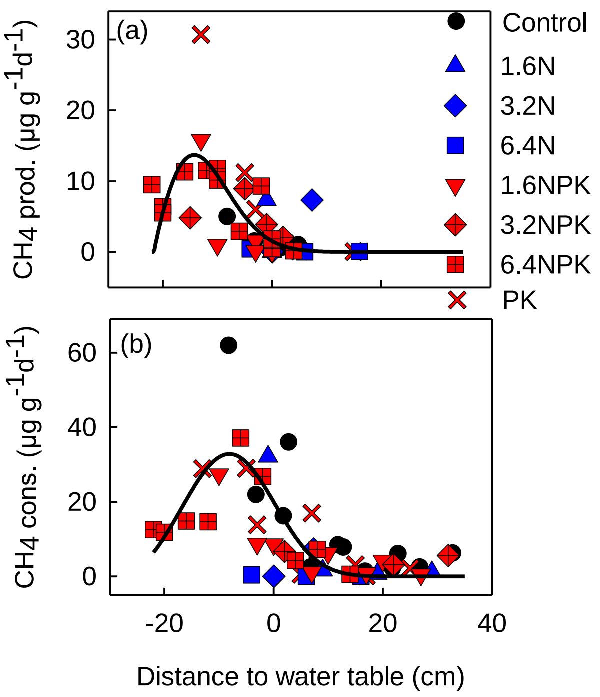

Supplementary information, Figure S1. Anaerobic CH_4 production (a) and consumption (b) potentials in relation to sample's distance to water table (WT) at the time of sampling. Negative WT values indicates position below the WT surface and positive values indicate position above the WT surface. To study the influence of a sample's distance to WT on potential CH_4 production and consumption, and to determine the optimal sampling depth for the second incubation study with the PK amendment, a Weibul function was fitted to the data. Replicates from the same peat section were averaged prior to running the analyses. Optimal depth was defined on the basis of where the potential CH_4 production or consumption reached its maximum.
